# Atropine as an Adjunct in the Management of Pediatric Patients With Hypertrophic Pyloric Stenosis: A Single Institution Experience and Review of the Literature

**DOI:** 10.7759/cureus.65363

**Published:** 2024-07-25

**Authors:** Charles Lu, John Marcin, Victoriya Staab

**Affiliations:** 1 Department of Pediatric Surgery, Hackensack Meridian Health Jersey Shore University Medical Center, Neptune, USA; 2 Department of Pediatric Surgery, Hackensack Meridian Health School of Medicine, Nutley, USA

**Keywords:** pediatric surgery, ramstedt, pyloromyotomy, pyloric stenosis, atropine

## Abstract

Infantile hypertrophic pyloric stenosis (IHPS) is a condition whereby there is a thickening of the pyloric muscle, leading to obstruction of the gastric outflow. Typically present within three to five weeks of life, it presents as postprandial non-bilious projectile vomiting. Commonly, a pyloromyotomy is the gold standard to relieve the obstruction. However, in a subset of patients not amenable to undergo surgery or anesthesia, or for postoperative persistent or recurrent obstruction, atropine may offer an alternative treatment. A retrospective review was performed on pediatric patients with hypertrophic pyloric stenosis utilizing the electronic medical record. Data included were demographics, workup data, treatment, outcomes, and symptom resolution. Approval was obtained by the institutional review board of the host institution. Five pediatric patients, with an average age of 2.1 months, received atropine treatment for IHPS. The average time to reach full feeds since the initiation of atropine was approximately four days. Three of the five patients were successfully managed with IV atropine, which was then transitioned to oral atropine and tapered off as outpatients, leading to the resolution of symptoms. The remaining two patients were considered failures of medical management and subsequently required surgery. Atropine use as an alternative treatment for IHPS may be considered when patients are not able to undergo surgery or anesthesia or have recurrent or persistent obstructive symptoms postoperatively. In this limited study, atropine was found to be safe and effective. Randomized controlled studies may lend additional merit to this therapy in the future.

## Introduction

Infantile hypertrophic pyloric stenosis (IHPS) is a condition in which the pyloric muscle becomes progressively abnormally thickened in a newborn, leading to gastric outlet obstruction within the first several weeks of life, specifically between three and five weeks. Several genetic and environmental factors are thought to contribute to this condition, including maternal smoking, bottle feeding, young maternal age, and erythromycin administration in the first two weeks of life [1]. It is a benign condition characterized by a projectile, non-bilious emesis in the newborn [2]. Ultrasound (US) is typically the gold standard method of diagnosis, but upper GI series can be helpful for diagnosis. Fluid and electrolyte correction of the hypochloremic, hypokalemic, and metabolic alkalosis is necessary to avoid postoperative apnea [2]. The treatment is typically a pyloromyotomy, which can be done either open or laparoscopically [2]. To our knowledge, very limited data exist addressing the utilization of atropine in patients with IHPS. The aim of this study was to present preliminary clinical outcomes associated with the use of atropine for IHPS.

## Case presentation

A retrospective review was performed on all patients who were admitted with a diagnosis of infantile pyloric stenosis between August 2016 and August 2023. A total of 121 patients were identified who were admitted with a diagnosis of pyloric stenosis. Five patients were identified who received atropine for a diagnosis of hypertrophic pyloric stenosis, utilizing the electronic medical record at a tertiary referral academic medical center. Data included patient demographics, treatment received, and outcomes (Table 1). The patients were diagnosed with US imaging (Table 2). Their admission labs were also reviewed (Table 3). The study was approved by the institutional review board at the host institution.

**Table 1:** Patient demographics HD, hospital day; RSV, respiratory syncytial virus

Patient	Age (weeks)	Sex	Surgery	Comorbidity	Atropine	Outcome
1	9	M	No surgery	Parainfluenza with neutropenia	Primary treatment	Resolution
2	5	F	Laparoscopic pyloromyotomy HD2	-	Postoperative	Resolution
3	4	M	Laparoscopic pyloromyotomy HD2	-	Postoperative	Recurrence of atypical symptoms, reoperation
4	5	M	No surgery	RSV	Primary treatment	Resolution
5	9	F	No surgery	RSV	Primary treatment	Surgery when symptoms persisted but RSV resolved

**Table 2:** Imaging UGI, upper gastrointestinal series; US, ultrasound

Patient	Imaging
1	US: 6 mm thickness, 20 mm length
2	US: 5.5 mm thickness, 17.5 mm length
3	US: 3.5 mm thickness, 18.4 mm length; UGI × 2 - no recurrence of IHPS, +delayed gastric emptying, + reflux; gastric emptying study: + for gastroparesis
4	US: 3 mm thickness, 19 mm length
5	US: 5 mm thickness, 21 mm length; repeat US three-month age: 6 × 21 mm

**Table 3:** Admission labs Cl, chloride; HC03, bicarbonate; K, potassium

Patient	Cl (mmol/L)	HCO3 (mmol/L)	K (mmol/L)
1	88	34	4.5
2	103	26	5.5
3	100	26	5.9
4	99	26	5.6
5	84	34	4.3

The five patients treated with atropine therapy initially received IV atropine while on a cardiac monitor, with no adverse events like tachycardia observed. The initial IV dose was 0.01 mg/kg administered 20 minutes before each feed, and it was gradually increased by 0.01 mg/kg until feed tolerance was achieved. Once enteral feeds were tolerated, patients were transitioned to the equivalent oral atropine dose and removed from cardiac monitoring. They were discharged after tolerating two full oral feeds without emesis, with a six-week outpatient weaning course to complete.

The treatment courses for the individual patients are as follows:

Patient 1

A nine-week-old male was treated nonoperatively for symptomatic parainfluenza. The length of stay (LOS) was seven days, with complete resolution of symptoms and no recurrence.

Patient 2

A five-week-old female underwent laparoscopic pyloromyotomy and developed recurrent obstructive symptoms nine days postoperatively. Instead of reoperation, she was treated with atropine, leading to symptom resolution. She was discharged on postoperative day 4 without evidence of a recurrence.

Patient 3

A four-week-old male had laparoscopic pyloromyotomy and developed recurrent obstructive symptoms six weeks later. He was hospitalized with *Klebsiella pneumoniae* and started on atropine therapy. A post-pyloric tube was placed temporarily. Despite a normal upper GI series, a delayed gastric emptying study revealed gastroparesis. He was discharged on oral atropine but required an open pyloromyotomy due to persistent vomiting and diarrhea, symptoms atypical for pyloric stenosis.

Patient 4

A five-week-old male was treated nonoperatively with atropine for symptomatic respiratory syncytial disease (RSV). Symptoms resolved, and he was discharged on postoperative day 9 without recurrence.

Patient 5

A nine-week-old female was treated nonoperatively for symptomatic RSV, with a nine-day LOS and resolution of symptoms. She developed recurrent symptoms during outpatient atropine therapy and subsequently underwent laparoscopic pyloromyotomy once RSV symptoms had resolved.

In summary, three patients were treated primarily with atropine due to a coinciding respiratory infection, which increased their risk for general anesthesia. Of these, two patients were successfully managed with atropine alone and did not require surgery. The third patient, who developed recurrent symptoms, had surgery after respiratory symptoms improved but continued to experience intermittent vomiting. Among the other two patients, one had postoperative obstructive symptoms that resolved completely with atropine. The remaining patient, who exhibited atypical delayed obstructive symptoms with normal imaging, underwent reoperation at seven months but still has gastrointestinal issues. The follow-up duration for these patients was eight years, and they are currently alive and well.

## Discussion

IHPS is a condition in which there is an abnormal hypertrophy of the pyloric muscle, resulting in the narrowing of the pyloric channel [3,4]. In infants, this may classically present as postprandial non-bilious projectile vomiting with or without a palpable “olive” on physical examination [3,4]. The diagnosis is further confirmed by US with the highest accuracy for diagnosis revealing a pyloric muscle thickness of at least 3.0 mm and a pyloric channel length of 14.5 mm [5]. The classic surgical procedure, first introduced by Ramstedt in 1912 describes an incision that extends from the vein of Mayo at the duodenal end to the circular fibers of the stomach proximally [6]. The classic surgical procedure, first introduced by Ramstedt in 1912, describes an incision that extends from the vein of Mayo at the duodenal end to the circular fibers of the stomach proximally [6]. This releases the narrowing of the pyloric channel, allowing for the passage of gastric contents. This can be done via an epigastric incision, a periumbilical incision, or laparoscopically. Specific surgical risks include airway complications such as laryngospasm or bronchospasm, aspiration, bleeding, wound infection, perforation of the myotomy, an incomplete myotomy, or recurrent obstruction.

Preoperative preparation is essential, as uncorrected metabolic derangements, typically hypochloremic or hypokalemic metabolic alkalosis, can result in postoperative apnea. Postoperatively, complications may include bleeding, wound infection, an incomplete pyloromyotomy, and missed perforation [3].

Conservative management for IHPS may be useful in that it does not have the associated surgical and anesthesia risks. Aseptic techniques with experts in pediatric surgery and pediatric anesthesia are not globally readily available, and that is why in many parts of the world, atropine therapy for IHPS has gained favorability. Treatment with atropine sulfate for IHPS has been studied in other countries, such as Japan, Serbia, Taiwan, Germany, and India, but little research exists on the efficacy of this medical treatment in the United States.

Prior studies have shown that pyloromyotomy has an overall success rate of approximately 95%, while for patients who may not be able to undergo surgery, atropine can provide an overall success rate of approximately 78.9% [7]. Thus, it may be reasonable to offer atropine to patients who are unfit to undergo general anesthesia due to medical comorbidities. The current literature that reflects the outcomes of atropine utilization ultimately demonstrates that there appeared to be an improvement in the thickness of the pylorus muscle, although it took longer for the atropine groups to reach full feeds [8-10]. There has, however, been a sparse amount of data published regarding the role of atropine use in HPS within the United States.

While the exact mechanism of atropine on the pylorus may not be well understood, it can be attributed to its role as a cholinergic blocking agent with potent antimuscarinic activity that decreases peristaltic contractions by relaxing the smooth muscle thought to contribute to muscular hypertrophy [8,9,11]. Additionally, it is thought that atropine is a strong inhibitor of meal-induced gastric acid secretion, limiting the acidic environment that may cause pyloric sphincter contraction [12]. The pharmacologic activity of IV atropine is approximately two to three times greater than that of the oral form, with potential side effects including transient tachycardia and flushing [13].

One study in Japan conducted in 2002 revealed that in 19 patients with infantile HPS, 17 patients ceased projectile vomiting after treatment with intravenous and subsequent oral atropine administration, with the remaining two infants requiring surgery [8]. Moreover, a 2005 study found that 45 out of 52 patients treated with a median of seven days of IV atropine and 44 days of oral atropine ceased vomiting and showed satisfactory recovery. The median hospital stay for these patients was 13 days (6-36) and involved no complications. Of the patients who received surgical intervention, four had wound infections, and one developed hemorrhagic shock. Thus, this study concluded that IV atropine has a high success rate and is an effective alternative to pyloromyotomy if the time to discharge and the continuation of oral atropine are deemed acceptable [8].

Furthermore, a 2005 study in India found that oral atropine was successful in 11 out of 12 cases (91.06%) of IHPS, with emesis ceasing in 14-21 days. The oral atropine was given at a dose of 0.18 mg/kg/day, increasing by ¼ of the first dose daily until vomiting stopped. One patient required IV atropine for seven days and oral atropine for 18 days. The pylorus was found to be normalized on US at three to 15 months in these patients. Therefore, this study concluded that oral atropine is a successful and cost-effective treatment for IHPS [9].

A meta-analysis by Wu et al. found that 77 of 110 (70%) of patients treated with oral atropine experienced induced remission of IHPS, 288 of 345 (83.5%) of patients treated with IV atropine then oral atropine showed satisfactory recovery with no deleterious side effects, and that pyloric muscle normalization was confirmed from five weeks to 15 months. Hence, this study concluded that atropine can be an effective alternative treatment for IHPS with few side effects, especially in patients with comorbidities [13]. However, a 2013 study from Lukac et al. found that 10 of 40 (25%) patients treated with atropine did not respond to therapy and still required surgical treatment, being released on days 3-5 following surgery, with the other 30 patients (75%) being released after vomiting ceased on days 6-8. There were no complications in either group. Thus, that study determined that there was a significant difference in favor of surgical treatment over atropine [14]. Additionally, a study from Meissner et al. established that a success rate of 75.8% (25/33) by day 7 of atropine treatment points towards the greater efficacy of surgical treatment of IHPS, which has a success rate of above 95% at day 7 [15].

Postoperative atropine use has rarely been described. Cubas et al. described 24 patients out of 965 reviewed who received postoperative atropine for emesis that persisted for more than 48 hours postoperatively. All the patients resolved their symptoms, with none returning to surgery [16]. Predictive factors for medical management failure have been described in two publications. Koike et al. found that projectile vomiting greater than five days after starting IV atropine is an indicator of failure of medical management and recommended considering early surgery [17]. Ono et al. demonstrated that age <30 days is an indicator of failure of management in 48 patients, 15 of whom needed surgery [18]. Adverse effects of rapid transient heart rate and flushing were described in two out of five patients who were treated with primary atropine therapy with a 100% success rate of symptom resolution [19].

In the present study, we sought to evaluate the role of atropine in our patients both as primary and as rescue therapy. We identified five patients with a diagnosis of IHPS who received atropine therapy over a seven-year retrospective review. While our patient pool is small, this study demonstrates that in select cases, either with recurrent obstructive symptoms after pyloromyotomy or for patients who are not surgical candidates at the time of presentation, a trial of atropine therapy can be used without any major adverse events.

Limitations of this study include the small sample size, which ultimately limits the power of the study. This makes the findings difficult to interpret; however, this does reflect the selective nature of this treatment option. The selection bias was our clinical decision in determining which patients were at high risk of primary surgical management based on their respiratory viral comorbidities. Additionally, this was a retrospective study and the duration of follow-up is limited to eight years. This study was not meant to test a hypothesis but rather demonstrate a novel clinical application of an innovative treatment option for patients not able to undergo pyloromyotomy.

## Conclusions

While our study reveals that atropine use may not be 100% effective, similar studies aforementioned report success rates lower than pyloromyotomy. Nonetheless, atropine may be a viable option for improving symptoms in patients who are not candidates for surgery or for those experiencing recurrent symptoms post-pyloromyotomy. We aim to highlight the potential role of atropine in managing these patients in the United States. This case series demonstrates our experience with atropine use in IHPS, but further studies involving a larger patient cohort are required to draw definitive conclusions. In this series, there were no short-term complications. Larger randomized prospective clinical studies are needed to provide additional insights regarding the safety and efficacy of atropine.
